# Increases in waist circumference independent of weight in Mongolia over the last decade: the Mongolian STEPS surveys

**DOI:** 10.1186/s40608-017-0155-3

**Published:** 2017-05-08

**Authors:** Oyun Chimeddamba, Emma Gearon, Samuel L. Brilleman, Enkhjargal Tumenjargal, Anna Peeters

**Affiliations:** 1 Department of Epidemiology and Preventive Medicine, School of Public Health and Preventive Medicine, Monash University, Level 6, 99 Commercial Road, Melbourne, VIC 3004 Australia; 2 Deakin University, Geelong, Victoria, School of Health and Social Development, Faculty of Health, Melbourne, Australia; 3 Victorian Centre for Biostatistics (ViCBiostat), Melbourne, VIC Australia; 4 Department of Health Development, National Center of Public Health, Peace Avenue 17, Bayanzurkh District-3, Ulaanbaatar, Mongolia

**Keywords:** Body mass index, Waist circumference, Abdominal obesity, Adults, STEPS, Mongolia

## Abstract

**Background:**

In Mongolia, mean waist circumference (WC) has increased dramatically over the last decade, however, it is unknown whether these increases have been greater than corresponding increases in weight. In this study we aimed to assess whether recent increases in WC were greater than expected from changes in weight in Mongolian adults.

**Methods:**

We used data on 13260 Mongolian adults, aged between 18 and 64 years, who participated in one of three (2005, 2009, 2013) nationally representative cross-sectional surveys. Linear regression was used to estimate changes in mean WC over time, adjusted for age, sex, height and weight. We also estimated the age-standardised prevalence for four obesity classification categories (not obese; obese by WC only; obese by body mass index (BMI) only; obese by both BMI and WC) at each survey year.

**Results:**

The estimated mean WC in 2009 and 2013, respectively, was 1.26 cm (95% CI: 0.35 to 2.17) and 1.88 cm (95% CI: 1.09 to 2.67) greater compared to 2005, after adjusting for age, sex, height and weight. Between 2005 and 2013, the age-standardised prevalence of those obese according to both BMI and WC increased from 8.0 to 13.6% for men and from 16.5 to 25.5% for women. During the same period, the percentage who were obese by WC only increased from 1.8 to 4.8% for men and from 16.5 to 26.8% for women. In contrast, the percentage who were obese by BMI only remained relatively stable (women: 2.4% in 2005 to 1.0% in 2013; men: 2.7% in 2005 to 4.0% in 2013).

**Conclusion:**

Over the last decade, among Mongolian adults, there has been substantially greater increase in WC and the prevalence of abdominal obesity than would be expected from increases in weight. Women are at greater risk than men of being misclassified as not obese if obesity is defined using BMI only. Obesity should be monitored using WC in addition to BMI to ensure the prevalence of obesity is not underestimated.

## Background

The prevalence of obesity in the Mongolian adult population appears to be increasing at a substantial rate [1–3]. In a previous study we have shown that, between 2005 and 2013, the age-standardised overall mean body mass index (BMI) and waist circumference (WC) of Mongolian adults increased by 1.4 kg/m^2^ and 5.2 cm, respectively [4]. During that same period the prevalence of general obesity, defined by BMI ≥ 30 kg/m^2^, increased from 10.8 to 17.6% for men and from 18.9 to 26.4% for women. The prevalence of substantially increased risk based on WC categories in men (9.5% in 2005 to 17.7% in 2013) and women (31.2% in 2005 to 50.6% in 2013) almost doubled between 2005 and 2013 [4].

Abdominal obesity as measured by WC may be a more accurate predictor of the metabolic risks of obesity compared to BMI [5–8]. For example, the Decoda Study Group in 2008 found that waist measurements were more strongly associated with diabetes than BMI [9]. In addition, a meta-analysis in 2013 examining the association between general and abdominal obesity measures and all-cause mortality suggested that WC was strongly associated with adult mortality, independent of BMI [6].

A number of recent studies in ethnically and geographically diverse populations have suggested that WC is increasing at a faster rate than weight [10–12]. For example, for Australian adults between 1995 and 2012, WC increased independent of increases in body weight by 6.7 cm (95% CI: 6.2 to 7.2) for women, and 2.8 cm (95% CI: 1.5 to 4.1) for men [13]. A study of American adults comparing trends in BMI and WC between 1988–1994 and 2005–2006 revealed an independent increase of 0.9 cm in mean WC over and above that for BMI [10]. Moreover, recently Albrecht et al., using four population-based surveys from the United States, England, China and Mexico demonstrated an increase in WC independent of BMI in all four countries, with the greatest independent increases observed among women aged 20–29 years in the rapidly developing countries of Mexico and China [14]. Further research conducted in China has also reported an increase in WC over time, measured as increases in mean WC within BMI categories, and the increases were again larger in younger women compared with older women [15].

Several other studies have also suggested that WC is increasing greater than BMI or body weight. For instance, a series of five nationally representative cross-sectional surveys of the United States population between 1959 and 2004 indicated that trivial and consistent increases in WC over time independently from BMI increase [16]. In Finnish men and women, mean WC increased by 2.7 cm and 4.3 cm in 15 years while mean BMI did not change much [17]. Lastly, central obesity measured by WC consistently increased in Chinese adult men between 1996 and 2005 while BMI remained unchanged [18]. However, there are few studies examining this in countries early in the nutrition transition. Changes in waist circumference independent of weight have not been investigated in the Mongolian context.

In the current study, we used repeated population-based cross-sectional surveys of Mongolian adults aged 18–64 years to estimate changes in mean WC over the past decade, independent of changes in weight. We also estimated changes in the age-standardised prevalence of four categories of obesity (not obese; obese by WC only; obese by BMI only; obese by both BMI and WC) in order to assess the potential for underestimating the increasing burden of obesity in Mongolian adults when only considering weight or BMI.

## Methods

### Sample

This study analysed data from the Mongolian STEPS Surveys on the prevalence of noncommunicable disease (NCD) risk factors, conducted in 2005, 2009 and 2013 [1–3]. The Mongolian STEPS surveys are a nationwide, nationally representative series of cross-sectional surveys conducted using the World Health Organization (WHO) NCD stepwise survey methodology with behavioural, anthropometric and biomedical measurements [19]. Participants were randomly selected using a four-stage cluster sampling design to cover all geographical areas of Mongolia. Identical sample design and weighting methodology were used at all three time points. Response rates in the respective surveys were 94.7% in 2005, 95.0% in 2009 and 97.4% in 2013. We excluded a small number of participants who had missing values for measured height, weight and waist circumference (26 in 2005; 125 in 2009; 150 in 2013) and, therefore, our analysis included 3102 participants in 2005, 5039 in 2009 and 5119 in 2013, respectively. In descriptive analyses we compared the demographic characteristics of those with and without missing data (Table 1). Each participant provided written informed consent and ethics approval to conduct the study was obtained from the Human Research Ethics Committee, Monash University on 1 April 2015 (CF15/1017 - 2015000474).

**Table 1:** Characteristics of survey participants with and without missing data in 2005, 2009 and 2013

	2005		2009		2013	
	Missing	No missing	Missing	No missing	Missing	No missing
Final sample size	26	3102	125	5039	150	5119
Age (Mean ± SD)	34 ± 12.3	40.9 ± 13.0	31.6 ± 8.8	38.3 ± 11.4	31.1 ± 9.3	37.3 ± 11.9
Sex (% Male)	46.1	48.5	17.2	41.2	14.8	45.2
Education (% Tertiary)	19.2	20.4	28.7	23.9	39.6	29.2
Employment (% Employed)	38.5	56.7	57.4	60.3	58.4	65.1
Geographical location (% Urban)	69.2	50.5	62.3	52.9	40.3	49.0

Abbreviation: *SD* standard deviation

### Anthropometric data

In all three surveys, trained personnel used a standardised protocol to collect anthropometric measurements. Weight was measured without shoes in light clothes on a lithium battery electronic weight scale with 0.1 kg precision and capacity of up to 100000 times measurements (Gima professional scale, Italy). Height was measured without shoes to the nearest 0.1 cm using a Somatometre-Stanley 04-116 device with the capacity to measure up to two meters, reading values in centimetres (cm). WC was measured in cm with a non-stretch tape measure to the nearest 0.1 cm at the mid-point between high point of the iliac crest and lower edge of the rib cage (Gima waist meter, Italy). Thus, anthropometric measurements were based on interviewer-measured height, weight and waist circumference rather than self-reported measurements.

### Demographic data

Age, sex, education status, and area of residence were also measured consistently at all three time points. Age was categorised as: 18-24, 25-34, 35-44, 45-54, and 55-64 years. Education was dichotomised into tertiary and non-tertiary based on each individual’s highest qualification. Area of residence was categorised as urban or rural.

### Statistical analyses

To describe the characteristics of the survey participants summary statistics were calculated based on unweighted data. In all other analyses we adjusted for the sample stratification and clustering effects and applied survey weights to individual responses to reflect the age, sex and geographical distribution of the Mongolian population at the time of each survey.

We defined obesity using several criteria. In our primary analyses we used the international thresholds: for BMI this was an obesity threshold of 30 kg/m^2^ (referred to throughout as “general obesity”) [20]; and for waist circumference we used the “substantially increased risk” threshold of ≥102 cm for men and ≥88 cm for women (referred to throughout as ‘abdominal obesity’) [21].

In 2004 a WHO expert consultation discussed BMI thresholds for determining overweight and obesity in Asian populations. This was deemed necessary since there is evidence that Asians are at a high risk of type 2diabetes and cardiovascular disease even at BMI values lower than the international thresholds. Although the expert consultation found there is no robust evidence that indicates clear overweight or obesity BMI thresholds for all Asians, the consultation did endorse public health action points (23.0, 27.5, 32.5, and 37.5 kg/m^2^) along with the international BMI thresholds [22]. Therefore, in secondary analyses we used Asian-specific thresholds: for BMI this was 27.5 kg/m^2^ (referred to throughout as “Asian-specific general obesity”) [22], and for waist circumference this was ≥102 cm for men and ≥88 cm for women (referred to throughout as “abdominal obesity”, noting that it reflects both international and Asian-specific thresholds) [23].

To describe changes in prevalence over time, we derived four categories for assessing obesity prevalence (example given here for the primary analysis, using international thresholds): not obese (BMI < 30, WC < 102 for men, WC < 88 for women), obese by WC only (BMI < 30, WC ≥ 102 for men, WC ≥ 88 for women), obese by both BMI and WC (BMI ≥ 30, WC ≥ 102 for men, WC ≥ 88 for women) and obese by BMI only (BMI ≥ 30, WC < 102 for men, WC < 88 for women). We estimated the age--standardised prevalence for each category at each survey year, both overall and stratified by sex (in primary and secondary analyses we considered each of the international and Asian-specific thresholds for defining obesity based on BMI, respectively). Age standardisation was conducted using the direct method [24] to eliminate the effect of differences in population age structures when comparing prevalence estimates across different periods of time and different geographical areas.

Linear regression was used to estimate changes in mean WC over time, with the survey year included as a categorical covariate (2005, 2009 and 2013) in the regression model. Model 1 was adjusted for sex, age group and height. Model 2 further adjusted for weight in addition to sex, age group and height. We also performed subgroup analyses to determine whether the changes in mean WC over time differed by sex, age group, area of residence or education status. To assess subgroup effects we fitted the linear regression described as Model 2, but also included one of the following pairwise interaction terms: sex and survey year; age group and survey year; area of residence and survey year; or education status and survey year. We assessed the importance of the interaction term using an F-test. All analyses were done using Stata version 14.0 (StataCorp LP, College Station, Texas, USA).

## Results

### Description of study participants

Table 1 summarises the demographic characteristics (unweighted) for the 13260 study participants who were included in the analysis. The mean age was comparable across survey years. There was a slight majority of female participants for all three surveys. The proportion of tertiary educated and employed participants increased at each survey. In 2013, around one third and two thirds of the sample were tertiary educated and employed, respectively. Approximately half of the participants in each survey resided in urban areas of Mongolia. Those with missing data (*n* = 301) were relatively younger, predominantly female, more tertiary educated, slightly less employed and more urban living individuals compared to those without missing data.

### Linear regression of waist circumference

Table 2 shows selected parameter estimates from each of the linear regression models; estimates shown in the table are the estimated difference in mean WC at each survey, compared to 2005. The mean WC increased over time, both before and after adjusting for changes in weight, with the regression coefficients smaller in magnitude in Model 2 (adjusted for weight) compared with Model 1 (not adjusted for weight). In Model 1, the estimated mean WC in 2009 and 2013, respectively, was 2.97 cm (95% CI: 2.01 to 3.93) and 4.93 cm (95% CI: 3.87 to 6.00) greater than in 2005. In Model 2, the estimated mean WC in 2009 and 2013, respectively, was 1.26 cm (95% CI: 0.35 to 2.17) and 1.88 cm (95% CI: 1.09 to 2.67) larger than in 2005.

**Table 2:** Parameter estimates (with 95% confidence intervals) for the change in mean WC over time

	Model 1^a^	Model 2^b^
Overall analysis (total sample)		
2009	2.97 (2.01 to 3.93)	1.26 (0.35 to 2.17)
2013	4.93 (3.87 to 6.00)	1.88 (1.09 to 2.67)
Subgroup analyses		
Sex		
Men		
2009	3.00 (1.56 to 4.43)	0.27 (-0.62 to 1.17)
2013	4.77 (3.18 to 6.37)	1.09 (0.39 to 1.80)
Women		
2009	3.01 (2.00 to 4.02)	2.32 (1.25 to 3.39)
2013	5.07 (4.00 to 6.14)	2.68 (1.64 to 3.71)
Age group (years)		
18–24		
2009	3.13 (1.85 to 4.42)	1.39 (0.37 to 2.42)
2013	3.74 (2.34 to 5.14)	1.75 (0.91 to 2.58)
25–34		
2009	3.35 (1.98 to 4.73)	1.53 (0.42 to 2.65)
2013	4.95 (3.74 to 6.16)	1.97 (1.09 to 2.84)
35–44		
2009	2.72 (1.32 to 4.13)	1.25 (0.36 to 2.15)
2013	4.90 (3.41 to 6.39)	1.88 (0.86 to 2.89)
45–54		
2009	1.98 (0.22 to 3.73)	0.65 (-0.49 to 1.79)
2013	5.09 (3.43 to 6.74)	1.63 (0.56 to 2.7)
55–64		
2009	3.49 (1.26 to 5.72)	1.25 (-0.08 to 2.57)
2013	4.82 (2.50 to 7.14)	1.94 (0.54 to 3.34)
Area of residence		
Urban		
2009	3.79 (2.25 to 5.33)	2.12 (0.88 to 3.35)
2013	5.83 (4.24 to 7.42)	2.42 (1.21 to 3.63)
Rural		
2009	2.21 (0.95 to 3.47)	0.43 (-0.95 to 1.81)
2013	4.08 (2.59 to 5.57)	1.37 (0.34 to 2.41)
Education		
Tertiary educated		
2009	2.05 (0.54 to 3.55)	0.80 (-0.59 to 2.19)
2013	3.56 (2.19 to 4.94)	1.86 (0.80 to 2.92)
Non-tertiary educated		
2009	3.18 (2.17 to 4.2)	1.40 (0.49 to 2.32)
2013	5.37 (4.19 to 6.54)	1.91 (1.09 to 2.74)

Abbreviations: *WC* waist circumference

^a^Model 1 regression model adjusted for age, sex, survey year and height

^b^Model 2 regression model adjusted for age, sex, survey year, height and weight

The coefficients shown in the table represent the estimated change in mean WC relative to 2005 (the reference category) Separate regression models were fit for each subgroup. The subgroup effects were estimated by including an interaction term between the relevant variable (sex, age group, education status or area of residence) and survey year. The model with the area of residence by survey year interaction effect also included the main effect for area of residence. Similarly, the model with the education status by survey year interaction effect also included the main effect for education status

The corresponding parameter estimates from subgroup analyses (by sex, age group, area of residence and education status) are also shown in Table 2. After adjusting for weight, there was evidence that the changes in mean WC over time independent of weight differed between men and women (F-test for the interaction term between sex and survey year: F-test (2, 75) =15.45, *p* <0.001). We found that women had greater increases in mean WC over time independent of weight (*β*_2009_ = 2.32, 95% CI: 1.25 to 3.39; *β*_2013_ = 2.68, 95% CI: 1.64 to 3.71) than men *β*_2009_ = 0.27, 95% CI: -0.62 to 1.17; *β*_2013_ = 1.09, 95% CI: 0.39 to 1.80). We found no evidence of an interaction between any of: age group and survey year (F-test (8, 69) =0.93, *p* = 0.50) or area of residence and survey year (F-test (2, 75) =1.37, *p* = 0.26). We found very weak evidence that the changes in mean WC over time independent of weight differed between tertiary and non-tertiary educated individuals (F-test (2, 75) =2.59, *p* = 0.08).

### Prevalence of obesity

Table 3 shows the age-standardised prevalence for each category of obesity using international thresholds at each survey year, both overall and stratified by sex. The overall prevalence of both general obesity (sum of ‘obese by BMI only’ and ‘obese by both BMI and WC’) and abdominal obesity (sum of ‘obese by WC only’ and ‘obese by both BMI and WC’) increased over time. The percentage who were ‘obese by WC only’ almost doubled between 2005 and 2013, from 8.9 to 15.9%, suggesting that an increasing number of individuals would be misclassified as not obese if we chose to only define obesity using BMI. The percentage who were ‘obese by both BMI and WC’ also increased, from 12.1% in 2005 to 19.6% in 2013. In contrast, the percentage who were ‘obese by BMI only’ remained at a similar level at all three time points (2.6% in 2005, 2.8% in 2009, and 2.5% in 2013).

**Table 3:** Age-standardised prevalence (%: with 95% confidence intervals) for each obesity category, as defined by international BMI and/or WC thresholds

	2005	2009	2013
Men			
Not obese	87.4 (84.6 to 89.8)	81.4 (77.9 to 84.5)	77.6 (75.2 to 79.9)
Obese by WC only	1.8 (1.1 to 3.0)	2.8 (2.0 to 4.0)	4.8 (3.7 to 6.3)
Obese by BMI only	2.7 (1.7 to 4.3)	4.5 (3.1 to 6.5)	4.0 (2.9 to 5.4)
Obese by both BMI and WC	8.0 (6.2 to 10.3)	11.2 (8.6 to 14.5)	13.6 (11.5 to 16.0)
Women			
Not obese	64.6 (61.6 to 67.5)	55.7 (52.6 to 58.6)	46.7 (43.6 to 49.9)
Obese by WC only	16.5 (13.6 to 19.8)	24.4 (22.0 to 27.0)	26.8 (23.3 to 30.7)
Obese by BMI only	2.4 (1.5 to 3.6)	1.1 (0.6 to 2.0)	1.0 (0.6 to 1.5)
Obese by both BMI and WC	16.5 (14.5 to 18.8)	18.8 (16.3 to 21.6)	25.5 (22.6 to 28.6)
Total			
Not obese	76.5 (74.6 to 78.3)	68.7 (66.3 to 71.0)	62.1 (59.6 to 64.6)
Obese by WC only	8.9 (7.4 to 10.6)	13.5 (12.0 to 15.0)	15.9 (13.6 to 18.4)
Obese by BMI only	2.6 (1.7 to 3.7)	2.8 (2.0 to 4.1)	2.5 (1.9 to 3.2)
Obese by both BMI and WC	12.1 (10.7 to 13.6)	15.0 (12.8 to 17.5)	19.6 (17.7 to 21.6)

Abbreviations: *WC* waist circumference, *BMI* body mass index

Prevalence estimates are represented as percentages

International BMI threshold for obesity is defined as BMI of 30 kg/m^2^ (general obesity)

International WC threshold for obesity is defined as the “substantially increased risk” threshold of ≥102 cm for men and ≥88 cm for women (abdominal obesity)

Not obese (BMI < 30, WC < 102 for men, WC < 88 for women), obese by WC only (BMI < 30, WC ≥ 102 for men, WC ≥ 88 for women), obese by both BMI and WC (BMI ≥ 30, WC ≥ 102 for men, WC ≥ 88 for women) and obese by BMI only (BMI ≥ 30, WC < 102 for men, WC < 88 for women)

In the analysis stratified by sex, the prevalence of both general and abdominal obesity was higher for women. Furthermore the percentage who were ‘obese by WC only’ was considerably greater for women compared to men. For example, in 2013, the age-standardised prevalence of individuals who were ‘obese by WC only’ was approximately five times greater for women (26.8%) compared to men (4.8%), suggesting that women would be at greater risk than men of misclassification (as non-obese) if obesity was defined using BMI only.

Table 4 shows the age-standardised prevalence for each category of obesity using the Asian-specific thresholds at each survey year, both overall and stratified by sex. The prevalence of general obesity was substantially higher when using the Asian-specific thresholds. For example, in 2013 the prevalence of Asian-specific general obesity was 38.3%, whilst the prevalence of general obesity based on international thresholds was 22.1%. When using Asian-specific thresholds, the prevalence of individuals who were classified as ‘obese by WC only’ was lower (for example, from 15.9% when using international thresholds to 7.2% when using Asian-specific thresholds, based on the estimates for 2013). However, overall the same trends were consistent with the international thresholds.

**Table 4:** Age-standardised prevalence (%: with 95% confidence intervals) for each obesity category, as defined by Asian specific BMI and/or WC thresholds

	2005	2009	2013
Men			
Not obese	79.7 (76.4 to 82.7)	68.4 (64.0 to 72.4)	66.4 (63.7 to 69.0)
Obese by WC only	0.3 (0.1 to 0.8)	1.0 (0.5 to 2.0)	0.8 (0.4 to 1.5)
Obese by BMI only	10.5 (8.5 to 12.9)	17.6 (14.9 to 20.7)	15.2 (13.1 to 17.6)
Obese by both BMI and WC	9.5 (7.5 to 12.0)	13.0 (10.4 to 16.2)	17.6 (15.0 to 20.5)
Women			
Not obese	59.5 (56.8 to 62.1)	52.1 (49.4 to 54.7)	42.8 (40.5 to 45.2)
Obese by WC only	7.1 (5.4 to 9.1)	12.4 (10.5 to 14.6)	13.5 (10.8 to 16.6)
Obese by BMI only	7.4 (5.4 to 10.2)	4.7 (3.4 to 6.5)	4.9 (3.5 to 6.8)
Obese by both BMI and WC	26.0 (23.5 to 28.6)	30.8 (27.8 to 34.0)	38.8 (36.0 to 41.7)
Total			
Not obese	70.0 (67.9 to 72.0)	60.3 (57.6 to 63.0)	54.6 (52.5 to 56.6)
Obese by WC only	3.5 (2.7 to 4.6)	6.6 (5.5 to 7.9)	7.2 (5.7 to 9.0)
Obese by BMI only	9.0 (7.4 to 11.0)	11.3 (9.4 to 13.5)	10.0 (8.6 to 11.6)
Obese by both BMI and WC	17.4 (15.7 to 19.3)	21.8 (19.6 to 24.2)	28.3 (26.1 to 30.5)

Abbreviations: *WC* waist circumference, *BMI* body mass index

Prevalence estimates are represented as percentages

Asian-specific BMI threshold for obesity is defined as BMI of 27.5 kg/m^2^ (Asian-specific general obesity)

Asian-specific WC threshold for obesity is defined as WC of ≥102 cm for men and ≥88 cm for women (Asian-specific abdominal obesity, noting that these thresholds are the same as the international thresholds)

Not obese (BMI < 27.5, WC < 102 for men, WC < 88 for women), obese by WC only (BMI < 27.5, WC ≥ 102 for men, WC ≥ 88 for women), obese by both BMI and WC (BMI ≥ 27.5, WC ≥ 102 for men, WC ≥ 88 for women) and obese by BMI only (BMI ≥ 27.5, WC < 102 for men, WC < 88 for women)

## Discussion

Over the last decade the Mongolian adult population has witnessed dramatic increases in mean WC [3]. In this study, we have shown that those increases in WC are even greater than what might be expected from the observed changes in weight. The increase in mean WC among Mongolian adults was, on average, 1.3 cm between 2005 and 2009 and 1.9 cm between 2005 and 2013, independent of changes in weight. We also observed that this increase in mean WC was greater for women compared to men. The age-standardised prevalence estimates for our four obesity classification categories suggest that the burden of obesity will be underestimated when classifying obesity using BMI alone.

We found that application of the Asian-specific BMI thresholds, while leading to a greater prevalence of general obesity than when using international BMI thresholds, did not affect our conclusions regarding changes in obesity prevalence over time. Overall, while the prevalence of abdominal obesity (obese by WC only) increased over time, the prevalence of general obesity (obese by BMI only) alone did not. We did, however, find that using Asian-specific thresholds reduced the potential for underestimating high risk adiposity when only considering BMI. Under Asian-specific thresholds, the percentage of women who were ‘obese by WC only’ was 13.5% in 2013. On the other hand, the percentage of males who were ‘obese by WC only’ when using Asian-specific thresholds was almost zero (0.8% in 2013).

Since there is evidence that Asians have higher body fat at lower BMIs and WCs compared to Western populations [25], we compared and contrasted our results with a similar ethnic population such as the Chinese. Data from a study of Hong Kong Chinese found that, between 1996 and 2005, WC had increased despite an apparent plateau in BMI [18].

Our results from subgroup analyses suggested that, compared with men, women had greater increases in mean WC independent of weight over time. Similar results have also been reported in other studies [12, 26, 27]. In contrast, a study by Stern et al. found that men and women had a 3.2 cm and 2.1 cm higher WC in 2009 compared to 1993, holding BMI constant [15].

We found some indication that increases in WC independent of weight were greater in those individuals who were non-tertiary educated compared with tertiary educated, which is a finding that has been reported in other studies [11, 26, 28]. However, the increases in WC over time did not differ between the various age groups or between individuals living in urban and rural areas.

Our study has several strengths. We used a series of large, nationally representative, cross-sectional STEPS surveys, which are part of an ongoing monitoring surveillance approach, known as the prevalence of NCD risk factors, established in Mongolia since 2005 with the WHO technical support. The use of consistent sampling methodology (a multi-stage random cluster process) meant that the sampling frame was comparable across all three time points. An equal distribution of study participants from urban and rural locations, and the high response rate, ensured the survey participants were representative of the entire country. A sufficient number of individuals allowed us to explore subgroup effects related to age, sex, area of residence and education in our analysis. Furthermore all three surveys used standardised measurements of weight, height and waist circumference, with all measurements taken by trained personnel. This means that the changes occurring in body weight and waist circumference were not attributable to systematic measurement error or differences in the measurement approach.

There are also some limitations with this study. Earlier commencement of the STEPS surveys could have provided more time points to inform trends over time. In addition, missing data was present in our analysis, albeit to a small degree. Nonetheless, cognisant that this missing data has the potential to lead to some bias in the estimates, we compared demographic differences between individuals with and without missing data. We found that those with missing data were relatively younger, predominantly female, more tertiary educated, slightly less employed and more urban living individuals compared to those without missing data. However, the small number of individuals (2.3%) with missing data means that these differences are unlikely to bias the estimates from our analyses. The findings of the current analysis are not a reflection of longitudinal changes in a single nationally representative cohort since the data analysed for this study were sourced from three cross-sectional surveys rather than based on repeated measures study design.

The key finding that WC is increasing at a faster rate than weight, suggests that changes in body fat distribution may be occurring in the Mongolian adult population. These potential changes in body fat distribution are a recently identified issue, particularly among Asian populations [29]. The causes of these changes remain unclear. Accumulated body fat stored in the abdominal region has been shown to be associated with a range of adverse health outcomes such as diabetes, metabolic syndrome, CVDs and all-cause mortality, independently of general obesity measured by BMI [30–34]. Evidence also suggests that a high WC is associated with lower muscle strength due to a higher accumulation of body fat [35].

One major implication of this study is that almost one in six persons in 2013 are classified as ‘obese by WC only’ and these individuals would not be detected as obese if they were screened using BMI measurements alone. Furthermore, we found the majority of these individuals were female and that the prevalence of this group is growing dramatically over time. Almost one-third of Mongolian adult women and around one-sixth if using Asian-specific BMI thresholds are likely to be classified as non-obese if we continue to screen by BMI alone. Similar findings have been reported in other Asian countries, for example, one study reported that two-thirds of Chinese adults who are classified as obese by WC would have been classified as normal weight if they were measured by BMI alone [26]. Studies comparing the metabolic health outcomes for “misclassified” individuals have been performed, and indicate that individuals with abdominal but not general obesity have increased metabolic risk to those with general obesity [36].

Obesity prevention is the key strategy for reducing the incidence of obesity-induced chronic diseases, however, the official government reporting from the Mongolian STEPS surveys documented the burden of obesity solely according to the international BMI thresholds [2]. Population-based monitoring of obesity in Mongolia inevitably needs to consider further adiposity measures, such as a combination of BMI and WC. If only BMI continues to be employed, a certain proportion of individuals will be undetected as obese and, thereby, more obesity-linked health consequences could be expected.

## Conclusions

In conclusion, our results indicate that, amongst Mongolian adults, both the mean WC and prevalence of abdominal obesity have increased substantially over the last decade. The estimated increases in WC appear greater than would be expected from increases in weight. Furthermore, about one in six Mongolian adults would be misclassified as not obese if we continue to define obesity exclusively using international BMI thresholds. Women are at greater risk than men of being misclassified as not obese if obesity is defined using BMI only. Obesity in Mongolia should be monitored using WC, in addition to BMI, to accurately capture secular trends in obesity and thereby improve the coverage of obesity prevention strategies, clinical intervention and public health monitoring.

## Abbreviations

BMI: Body mass index
NCD: Noncommunicable disease
STEPS: Stepwise approach to chronic disease risk factor surveillance
WC: Waist circumference
WHO: World Health Organization
